# Duodenal gangliocytic paraganglioma: A very rare cause for upper gastrointestinal bleeding: Case report with review of literature

**DOI:** 10.1016/j.ijscr.2020.09.129

**Published:** 2020-09-23

**Authors:** Sardar Hassan Arif, Ayad Ahmad Mohammed, Rafil T. Yaqo

**Affiliations:** a Department of Surgery, College of Medicine, University of Duhok, Kurdistan Region, Iraq; b Department of Pathology, University of Duhok, Kurdistan Region, Iraq

**Keywords:** Gangliocytic paraganglioma, Gastrointestinal bleeding, Spindle cells, Epithelial cells, Ganglion cells

## Abstract

**Introduction:**

Gangliocytic paraganglioma is a rare tumors mainly arises from the second part of the duodenum. The tumor is generally benign, but some cases are associated with lymph node or distant metastasis. It must be differentiated from other tumors. The main treatment is good local surgical resection. Most patients have good prognosis after an appropriate resection.

**Case presentation:**

A 47-year-old male patient presented with recurrent attacks of epigastric pain and melena. The general examination revealed pallor with no jaundice. The abdomen was soft with no tenderness. There were no stigmata of chronic liver diseases. The hemoglobin level was 8 gm/dl with normal liver and renal functions. The patients received compatible blood. Endoscopy showed a large pedunculated polyp with ulceration in the second part of the duodenum just distal to ampulla of Vater. During surgery, the anterior wall of duodenum was opened, and a 4 cm pedunculated polyp was excised which was arising from the posterior wall of the duodenum just distal to the ampulla of Vater. The frozen section examination revealed a benign lesion. The final histopathology result was consistent with the diagnosis of gangliocytic paraganglioma. The patient had smooth post-operative recovery and no postoperative complications.

**Conclusions:**

Gangliocytic paraganglioma is a rare tumor which mostly affects the second part of the duodenum. The tumor is considered benign but the possibility of the malignancy with lymphatic and distant metastases must be excluded before any surgery. The management plan must be addressed carefully and continuous follow up is recommended.

## Highlights

• Gangliocytic paraganglioma is an exceedingly rare tumors that is mainly arises from the second part of the duodenum.
• The tumor was first described by Dahl et al. in 1957.
• The tumor has a characteristic 3 distinct types of cells: spindle cells, epithelial cells, and ganglion cells.
• The tumor must be differentiated from other tumors.
• Although it is a benign tumor but some cases have lymphatic or distant organ metastases.

## 1 Introduction

Gangliocytic paraganglioma (GP) is an exceedingly rare tumors that is mainly arises from the second part of the duodenum in close proximity to the ampulla of Vater, although the tumor can be seen throughput the gastrointestinal tract [[1], [2], [3]].

The tumor is generally classified as a benign tumor, but some cases are reported which are associated with lymph node metastases or even distant metastases in some rare cases [1,3].

The tumor was first described by Dahl et al. in 1957, after that some cases were reported until 1971 when Kepes and Zacharias named the tumor as *gangliocytic paraganglioma* based on the common features of both paraganglioma and ganglioneuroma. Kepes and Zacharias also described its characteristics cellular features. The tumor has a characteristic 3 distinct types of cells: spindle cells, epithelial cells, and ganglion cells, identification of all these three components is essential for the diagnosis [1,3].

The tumor usually affects middle aged people with slight male predominance. There is no specific symptom related to the tumor, patients may be asymptomatic and discovered incidentally during imaging for other purposes, it may cause mass effect causing abdominal pain, gastrointestinal bleeding which could be life threatening due to mucosal ulceration over the lesion, features of intestinal obstruction or bile duct obstruction. Most cases are hormonally inactive, however, some cases are reported with somatostatin or pancreatic polypeptide secretion. One case is reported in association with Von Recklinghausen disease suggesting a possible link [4,5].

This tumor have a variety of differential diagnoses such as carcinoid tumor, ganglio-neuroma, pigmented paraganglioma and gastrointestinal stromal tumor (GIST), hamartoma, lymphoma, hemangioma and duodenal cancer and sarcomas. This differentiation is usually done by imaging, biopsy or immunohistochemical analyses detecting the expression of various markers and proteins [1,[4], [5], [6], [7], [8]].

The accurate preoperative diagnosis is very difficult in most cases. The main treatment is good local surgical resection either by the open approach or endoscopic approach, most patients have good prognosis after an appropriate resection [3].

The work of this report case has been reported in line with the SCARE 2018 criteria [9].

## 2 Patient information

A 47-year-old male patient presented to the surgical consultation with recurrent attacks of epigastric pain, melena, and epigastric fullness.

Upper and lower GIT endoscopies were performed during the first admission which showed no abnormal findings.

During the last presentation, the patient was admitted to the surgical unit.

The patients had negative drug history. The family history was negative for any genetic diseases, and the psychosocial history was negative.

### 2.1 Clinical findings

The general examination revealed pale conjunctivae, palms and oral mucosa. There were no cyanosis or jaundice.

The blood pressure was 100/60 mmHg on admission, the pulse rate was 95 beats/minute, the temperature was normal and the body mass index was 38.

The abdomen was soft with no areas of tenderness. There were no dilated veins over the abdomen with no stigmata of chronic liver diseases.

### 2.2 Diagnostic assessment

Investigations were performed for the patient. The hemoglobin level was 8 gm/dl with normal serum electrolytes, normal liver and renal function tests. The patients received 2 units of compatible blood and arrangement was done to perform another endoscopy.

**Fig. 1:**
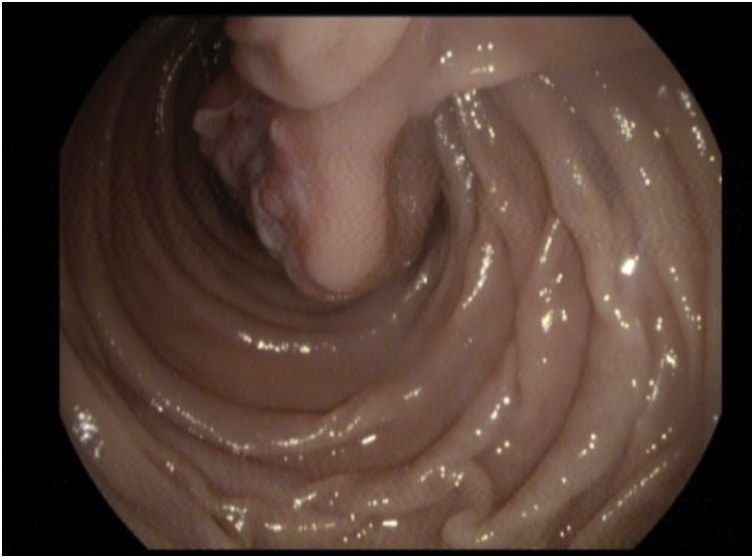
An endoscopic picture showing the pedunculated polyp arising from the 2nd wall of the duodenum.

The endoscopy this time revealed a large pedunculated polyp with ulceration in the second part of the duodenum just distal to ampulla of Vater. Fig. 1.

CT-scan of the abdomen was normal with no abnormal findings.

### 2.3 Therapeutic intervention

Arrangement was done to perform surgical intervention, during surgery we adopt an upper midline incision, exploration of the abdominal cavity showed normal liver, spleen, and omentum, with no ascites and no dilated veins.

**Fig. 2:**
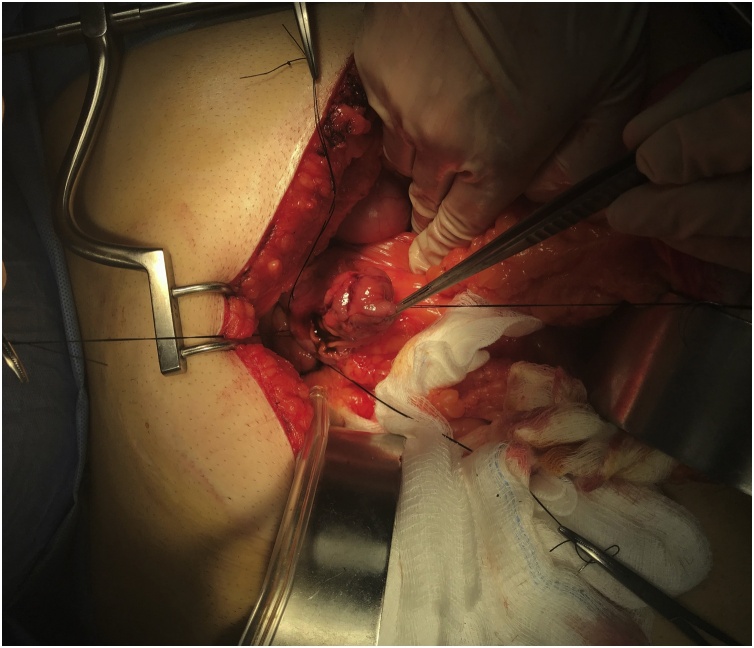
An intraoperative picture showing the polyp after anterior duodenotomy.

**Fig. 3:**
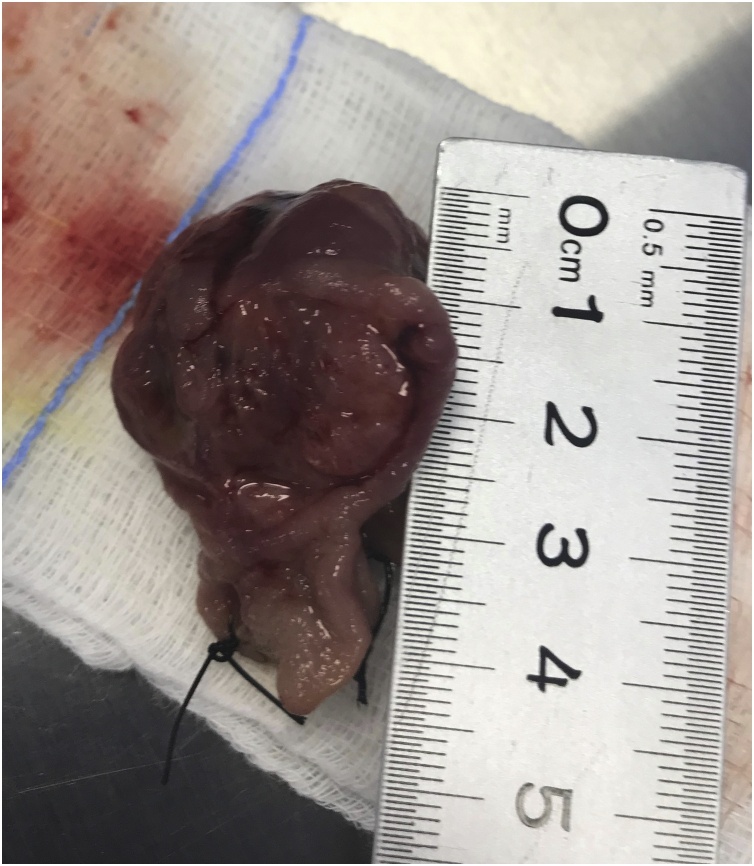
Showing the 4 cm polyp after being extracted.

Mobilization (kocherization) of the duodenum was performed, the anterior wall of duodenum was opened, and a pedunculated polyp (about 4 cm in size) was found which was arising from the posterior wall of the duodenum just distal to the ampulla of Vater. Fig. 2, Fig. 3.

Excision of the polyp with its base was performed and was sent for frozen section examination during surgery which revealed a benign lesion with no signs of malignant activity.

The posterior and the anterior duodenal walls were sutured transversely in two layers by a slowly absorbable 3/0 suture material (polydioxanone). The inner layer with continuous full thickness suturing and the outer layer with interrupted seromuscular suturing.

The procedure was performed by two general surgeons who are specialized in the field of general surgery.

**Fig. 4:**
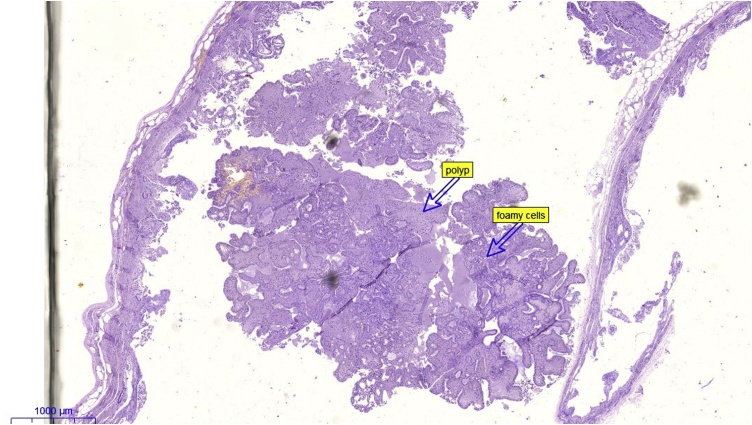
A microscopic picture showing the polyp arising from the duodenal wall.

**Fig. 5:**
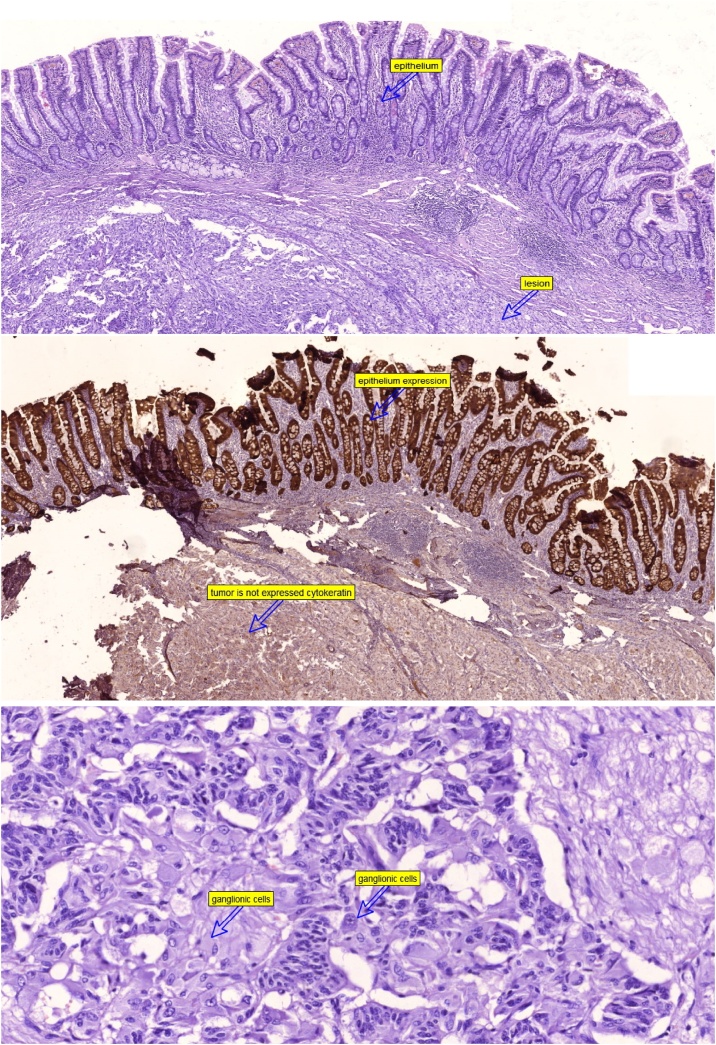
A microspic picture showing: a) the tumor lined by the duodenal epithelium, b) The negative staining of the tumor cells for the cytokeratin and the positive staining of the epithelial layer for the cytokeratin, c) The ganglionic and the paraganglionic cells.

The final histopathology examination revealed a well-defined mass in the submucosa of the duodenum that showed tri-phasic endocrine components; i.e. carcinoid like cells in compact nests and trabeculae, spindle cells (Schwan cells), and ganglion type cells of varying proportions. The immunohistochemical analysis of the polyp showed that all cells stained positive for synaptophysin, Schwan cells stained positive for S100 marker, with focal expression of pankerain in the endocrine cells. The mucosa and the margins were free from the tumor. These features were consistent with the diagnosis of duodenal Gangliocytic paraganglioma. Fig. 4, Fig. 5.

### 2.4 Follow-up and outcomes

The patient was admitted to the hospital for 5 days with smooth post-operative recovery and no postoperative complications.

No specific postoperative considerations were undertaken.

## 3 Discussion

The mean size of the reported cases was 25 mm size with maximum reported size of 10 cm. Tumors are usually is well demarcated with no definitive capsule [4].

Immunohistochemical study is very important in the diagnosis, for epithelial cells and ganglion like cells CD56 and Syn show highest positive rates, while spindle-shaped cells express high levels of S-100 protein. It is reported that epithelioid and ganglion cells stain positive to the neuroendocrine peptides like somatostatin, pancreatic polypeptide, and serotonin [1,5].

The incidence of lymph node metastases may be underestimated, this this is because most cases are treated with local excision without lymphatic dissection [4].

There are less than 30 reported cases with lymphatic metastases and few cases with distant organ metastases, two cases of liver metastases and one with bone metastases have been reported. The malignant potential of the tumor depends on the size of the tumor being higher when the tumor is larger than 2 cm, younger age of the patient, and tumors invading the submucosa. Some authors suggest that tumors expressing bcl-2 and/or p53 also increase the metastatic potential to the regional lymphatics or distant organs [4,10].

The possible treatment options varies greatly depending on the clinical presentation, the size of the tumor and the local extension. Some cases are treated by endoscopic polypectomy especially in pedunculated lesions, the depth of the tumor invasion must be determined by endoscopic ultrasound before resection is performed. Simple excision is recommended as a curative management in some cases, other treatment option is pylorus preserving pancreaticoduodenectomy with dissection of the regional lymph nodes [2,4].

The management options must be discussed and planned carefully after an appropriate preoperative staging whenever possible but this may not be applicable especially in emergency presentations. Some patients may present with massive upper GIT bleeding and in such cases the priority is for resuscitation and emergency surgery may be required. Tumors that have an aggressive behavior and have local or distant invasion are treated with adjuvant radiation therapy. Continuous follow up is recommended due to the malignant potential in some cases [5,10,11].

## 4 Conclusion

Gangliocytic paraganglioma is a unique type of tumor which mostly affects the second part of the duodenum. Although the tumor is considered benign in most literature, but the possibility of the malignant potential with lymphatic and distant organ metastases must be kept in mind and must be excluded before any form of surgery. The management is plan must be addressed carefully and continuous follow up is recommended.

## Declaration of Competing Interest

The authors report no declarations of interest.

## Funding

None.

## Ethical approval

Ethical approval has been exempted by my institution for reporting this case.

## Consent

An informed written consent was taken from the patient for reporting the case and the accompanying images.

## Author contribution

Dr Ayad Ahmad Mohammed and Dr Sardar Hassan Arif contributed to the concept of reporting the case and the patient data recording.

Drafting the work, design, and revision done by Dr Ayad Ahmad Mohammed.

Dr Rafil T. Yaqo did the histopathological examination and immunohistochemical analyses.

Final approval of the work to be published was done by Dr Ayad Ahmad Mohammed and Dr Sardar Hassan Arif.

## Registration of research studies

This work is case report and there is no need of registration.

## Guarantor

Dr Ayad Ahmad Mohammed is guarantor for the work.

## Provenance and peer review

Not commissioned, externally peer-reviewed.
